# Y-box binding protein-1 serine 102 is a downstream target of p90 ribosomal S6 kinase in basal-like breast cancer cells

**DOI:** 10.1186/bcr2202

**Published:** 2008-11-27

**Authors:** Anna L Stratford, Christopher J Fry, Curtis Desilets, Alastair H Davies, Yong Y Cho, Yvonne Li, Zigang Dong, Isabelle M Berquin, Philippe P Roux, Sandra E Dunn

**Affiliations:** 1 Laboratory for Oncogenomic Research, Department of Pediatrics, Child and Family Research Institute, University of British Columbia, Vancouver, BC V5Z 4H4, Canada; 2 Cell Signaling Technology, 3 Trask Lane, Danvers, MA 01923, USA; 3 Hormel Institute, University of Minnesota, 801 16th Avenue NE, Austin, MN 55912, USA; 4 Department of Cancer Biology, Wake Forest University School of Medicine, Medical Center Boulevard, Winston-Salem, NC 27157, USA; 5 Department of Pathology and Cell Biology, Faculty of Medicine, Institute for Research in Immunology and Cancer, P.O. Box 6128, Station Centre-Ville, Université de Montréal, Montreal, QC H3C 3J7, Canada

## Abstract

**Introduction:**

Basal-like breast cancers (BLBC) frequently overexpress the epidermal growth factor receptor (EGFR) and subsequently have high levels of signaling through the MAP kinase pathway, which is thought to contribute to their aggressive behavior. While we have previously reported the expression of Y-box binding protein-1 (YB-1) in 73% of BLBC, it is unclear whether it can be regulated by a component of the MAP kinase signaling pathway. Phosphorylation of YB-1 at the serine 102 residue is required for transcriptional activation of growth-enhancing genes, such as EGFR. Using Motifscan we identified p90 ribosomal S6 kinase (RSK) as a potential candidate for activating YB-1.

**Methods:**

Inhibition of RSK1 and RSK2 was achieved using siRNA and the small molecule SL0101. RSK1, RSK2, activated RSK and kinase-dead RSK were expressed in HCC1937 cells. Kinase assays were performed to illustrate direct phosphorylation of YB-1 by RSK. The impact of inhibiting RSK on YB-1 function was measured by luciferase assays and chromatin immunoprecipitation.

**Results:**

Using an *in vitro *kinase assay, RSK1 and RSK2 were shown to directly phosphorylate YB-1. Interestingly, they were more effective activators of YB-1 than AKT or another novel YB-1 kinase, PKCα. Phosphorylation of YB-1 (serine 102 residue) is blocked by inhibition of the MAP kinase pathway or by perturbing RSK1/RSK2 with siRNA or SL0101. In immortalized breast epithelial cells where RSK is active yet AKT is not, YB-1 is phosphorylated. Supporting this observation, RSK2^-/- ^mouse embryo fibroblasts lose the ability to phosphorylate YB-1 in response to epidermal growth factor. This subsequently interfered with the ability of YB-1 to regulate the expression of EGFR. The RSK inhibitor SL0101 decreased the ability of YB-1 to bind the promoter, transactivate and ultimately reduce EGFR expression. In concordance with these results the expression of constitutively active RSK1 increased YB-1 phosphorylation, yet the kinase-dead RSK did not.

**Conclusions:**

We therefore conclude that RSK1/RSK2 are novel activators of YB-1, able to phosphorylate the serine 102 residue. This provides a newly described mechanism whereby YB-1 is activated in breast cancer. This implicates the EGFR/RSK/YB-1 pathway as an important component of BLBC, providing an important opportunity for therapeutic intervention.

## Introduction

Basal-like breast cancers (BLBC) are clinically challenging cases that are not amenable to current targeted therapies due to the absence of estrogen receptor or HER-2 expression. Treatment therefore depends on aggressive chemotherapy, yet relapse rates and overall survival are poor. Identification of potential therapeutic targets is an ongoing challenge.

Y-box binding protein-1 (YB-1) is an oncogenic transcription/translation factor that is overexpressed in a number of cancer types, including breast cancer [1,2], prostate cancer [3], bone cancer [4], lung cancer [5,6], colon cancer [7], muscle cancer [8] and, most recently, pediatric brain tumours [9]. In particular, we have shown YB-1 to be expressed in a high proportion of BLBC [1], where it is associated with high rates of relapse [10]. Overexpression of YB-1 in breast cancer cells results in an increase in monolayer and enhanced anchorage independent growth [11]. Further, a study by Bergmann and colleagues demonstrated that targeted expression of YB-1 in the mammary gland of mice resulted in tumour formation with 100% penetrance [12]. Conversely, we find that suppressing YB-1 using RNA interference inhibits tumour cell growth *in vitro *[1] and *in vivo *[13]. The role of YB-1 in promoting growth of breast cancer cells stems from its original identification as a DNA binding protein, interacting with the regulatory elements of epidermal growth factor receptor (EGFR), HER-2 [14] and c-MYC [15].

In the succeeding 20 years since these findings, many more growth-promoting genes have been identified as YB-1 targets, including topoisomerase II [7], DNA polymerase alpha and proliferating cell nuclear antigen (PCNA) [16] to name just a few examples. The question that arises is how YB-1 becomes activated to induce the expression of these genes so central to the development of cancer.

We previously demonstrated the importance of phosphorylation at the serine 102 residue (S102) to the functions of YB-1 [1,2]. This site lies in the highly conserved cold-shock domain and is key for YB-1 nuclear localization and its ability to transform cells [11]. Recent studies have provided evidence for the vital role of phosphorylation this residue plays in the binding of YB-1 to, and the regulation of, the EGFR promoter and subsequent protein production [1,2]. In short, we have shown MCF-7 breast cancer cells overexpressing YB-1 have elevated levels of EGFR mRNA and protein [2]. Subsequently we reported that YB-1 bound the EGFR promoter in BLBC cells in a S102 phosphorylation-dependent manner [1]. Several studies have also implicated the importance of S102 phosphorylation in promoting translation [17,18]. Phosphorylation of S102 is therefore important for activating the transcriptional and translational control imparted by YB-1.

We previously demonstrated that AKT binds directly to YB-1 and phosphorylates the S102 site [11], an observation subsequently confirmed in NIH3T3 cells [18]. A recent study by Basaki and colleagues showed that serum stimulated YB-1 nuclear localization in ovarian cells and, further, this translocation was prevented by inhibiting AKT [19].

The Phosphatidylinositol-3 kinase (PI3K) pathway may not be the major contributor to growth in BLBC. EGFR is expressed in at least 50% of BLBC [20] and was recently used as one of five markers to identify aggressive BLBC [21]. We previously found that, by inhibiting EGFR with Iressa, we could slow the growth of BLBC cells [1]. Since this receptor signals through the MAP kinase pathway, we questioned whether other kinases are able to phosphorylate this key residue. We therefore took a bioinformatics approach to identify potential candidates, and determined that p90 ribosomal S6 kinase (RSK) may also phosphorylate YB-1 at S102 [22]. RSK1 to RSK4 are members of the AGC serine/threonine superfamily of kinases [23] that lie downstream of the MAP kinase pathway. RSKs are a direct substrate of ERK [24], but also require phosphorylation by phosphoinositide-dependent protein kinase-1 (PDK-1) [25] and subsequent autophosphorylation steps [26].

The importance of RSK family members in diseases such as cancer is just being appreciated. Of the four isoforms, RSK1 and RSK2 are the most well characterized, and overexpression has been associated with multiple cancer types such as prostate cancer [27] and those of hematologic malignancies [28]. Recent studies showed that RSK3 may actually be a tumour suppressor in ovarian cancer [29], and RSK4 differed from the other isoforms in that it was expressed at low levels and was constitutively active [30]. In breast cancer, a small study carried out by Smith and colleagues found that both RSK1 and RSK2 expression levels were elevated in ~50% of tumours compared with control cases (n = 12 controls, n = 48 cancers) [31]. We questioned whether RSK1 or RSK2 may play a role in BLBC because they lie in the MAP kinase pathway, which is commonly activated in this type of breast cancer due to overexpression of EGFR. In light of studies showing that RSK phosphorylates other transcription factors such as creb, c-fos [32] and the estrogen receptor [33], we contended that it may play an important role in regulating YB-1.

## Materials and methods

### Cell lines and reagents

The SUM149, HCC1937, MDA-MB-231 and MDA-MB-468 cells were used as models of BLBC; all are estrogen receptor negative, progesterone receptor negative and HER-2 negative [34]. SUM149 cells were purchased from Asterand (Ann Arbor, MI, USA) and were cultured as previously described [1]. MDA-MB-231 and MDA-MB-468 (both American Type Culture Collection, Manassas, VA, USA) cells were grown in DMEM (Gibco/Invitrogen, Burlington, ON, Canada) supplemented with 10% FBS and 100 units/ml penicillin/streptomycin. HCC1937 cells (kind donation from WD Foulkes, McGill University, QC, Canada) were cultured in RPMI-1640 media supplemented with 5% FBS, 10 mM HEPES, 4.5 g/l glucose (Sigma, Oakville, ON, Canada), 1 mM sodium pyruvate (Sigma) and 100 units/ml penicillin/streptomycin.

HTR-YB#5 (HTRY) are human mammary epithelial cells immortalized with HPV16, and express YB-1 if induced with tetracycline [35]. These were maintained in the same media as SUM149 cells supplemented with 10 ng/ml epidermal growth factor (provided by author IMB). RSK1/RSK2 specific inhibitor SL0101 (Toronto Research Chemicals Inc., North York, ON, Canada) was dissolved in methanol [31,36,37], and PD098059 (Cell Signaling Technologies, Danvers, MA, USA), phorbal 12-myristate 13-acetate (PMA) (Sigma) and epidermal growth factor (EGF) were dissolved in dimethylsulfoxide (DMSO).

### Growth factor stimulation and drug treatments

SUM149 cells were seeded at a density of 4 × 10^5 ^cells in a six-well plate. Cells were subsequently serum-starved for 24 hours prior to 6 hours treatment with vehicle, PD098059 (20 μM) or SL0101 (50 μM). Treated cells were stimulated with the following growth factors for 15 minutes before harvesting; 5% FBS/Ham's/F12 (serum stimulation), EGF (25 ng/ml) and PMA (50 ng/ml), lysed in egg lysis buffer (ELB) and subjected to western blot analysis [2]. In all other experiments, HCC1937, MDA-MB-231 and HTRY cells were treated with 100 μM SL0101 and the SUM149 cells with 50 μM for 6 hours. The experiment was performed three times.

### Protein extraction and western blot analysis

Protein was extracted from log-growing cells in ELB [2], supplemented with protease and phosphatase inhibitors, and quantified using the Bradford assay (Biorad, Hercules, CA, USA). Immunoblotting was performed as previously described [2]. Specific proteins were detected using the following antibodies: EGFR, 1:1,000 (Stressgen, San Diego, CA, USA); ERK, 1:1,000 (p44/42 MAP kinase; Cell Signaling Technology, Danvers, MA, USA); RSK1, 1:1,000 (Santa Cruz Biotechnology, Santa Cruz, CA, USA); RSK2, 1:500 (Santa Cruz Biotechnology); YB-1, 1:2,000 (Abcam, Cambridge, MA, USA); P-ERK, 1:500 (Cell Signaling Technology); P-RSK^S380^, 1:1,000 (Cell Signaling Technology); P-YB-1^S102^, 1:1,500 (Cell Signaling Technology, Danvers, MA, USA); Vinculin, 1:1,000 (Upstate, Temecula, CA, USA); and Pan-actin, 1:1,000 (Cell Signaling Technology). Densitometry was performed where appropriate.

### RSK/AKT kinase assay

A synthetic peptidomimetic of the YB-1 S102 region was manufactured by Sigma with the sequence PRKYLRSVG-COOH. Kinase assays for RSK1, RSK2, AKT1 and PKCα were carried out on the peptide and activity was compared with an optimized control target (100% activity) (SignalChem, Richmond, BC, Canada). Control target sequences were as follows: RSK, KRRRLASLR; AKT1, CKRPRAASFAE; PKC, KRREILSRRPSYR.

The kinase assay reactions consisted of active protein kinase (250 ng/assay), substrate (optimized, YB-1 peptide or assay buffer) (40 μM), radiolabeled ^33^P-ATP (50 μM in kinase assay buffer; 25 mM MOPS, 12.5 mM β-glycerol phosphate, 25 mM MgCl_2_, 5 mM ethylene glycol tetraacetic acid (EGTA), 2 mM ethylenediamine tetraacetic acid, 0.25 mM dithiothreitol) to a final volume of 25 μl. Assays were performed at 30°C for 60 minutes, and then the reaction mixture was dotted on phosphocellulose P81 paper and the radioactivity measured. Activity greater than 5% of the optimized positive control is considered highly significant.

### RSK1/YB-1 kinase assay from cell lysates

MCF-7 cells stably expressing Flag-YB-1 were serum starved for 16 hours prior to being lysed in radio immunoprecipitation assay (RIPA) buffer. As described above, 500 μg lysate was precleared with protein G agarose for 2 hours. YB-1 was then immunoprecipitated from the cells by overnight incubation at 4°C with 5 μg anti-Flag M2 antibody (Sigma) followed by 2 hours of incubation with protein G agarose. Complexes were then collected by centrifugation and washed firstly in Tris-buffered saline/1% NP40 and then once in modified wash buffer (100 mM Tris, pH 7.4, 50 mM NaCl, 1.5 mM MgCl_2_, 1 mM ethylenediamine tetraacetic acid, 0.5% NP40). YB-1 was isolated from protein G through incubation in 0.1 M glycine, pH 3.5, for 5 min at room temperature. Kinase assays were performed for RSK1 as described above.

### Co-immunoprecipitation

Log-growing SUM149 cells were lysed in RIPA buffer supplemented with protease inhibitors. Cell lysates were subjected to a Bradford assay for quantification and 500 μg protein was used in subsequent immunoprecipitations. For YB-1 pull-down, lysates were precleared with 60 μl PrecipHen beads (previously described [2]) for 2 hours at 4°C with rotation, and the supernatants then incubated with IgY or chicken anti-YB-1 antibodies (5 μg) overnight at 4°C with rotation. Immunocomplexes were collected on PrecipHen beads after incubation at 4°C for 3 hours, by centrifugation. The beads were washed once with PBS/1% NP40, twice with wash buffer (100 mM Tris, pH 7.4, 100 mM NaCl, 1.5 mM MgCl_2_, 1 mM ethylenediamine tetraacetic acid, 0.5% NP40) and the proteins eluted by boiling in 5× loading dye for 5 minutes.

Similarly, for total RSK1/RSK2 immunoprecipitations, lysates (500 μg) were precleared with 35 μl protein G agarose for 2 hours prior to incubation with either control IgG or RSK1 or RSK2 antibodies (5 μg) (Santa Cruz Biotechnology) for 16 hours at 4°C with rotation. Immunocomplexes were retrieved through the addition of protein G agarose for 2 hours. Immunoprecipitated proteins were resolved on acrylamide gels and immunoblotted as described above. Horseradish peroxidase protein A was used as the secondary antibody to avoid detection of denatured immunoglobulins (1:2,000; Amersham Biosciences, Piscataway, NJ, USA).

### RSK1 and RSK2 siRNA transfection

SUM149 cells (4 × 10^5^/well) were transfected with 20 nM siRNA (Qiagen, Mississauga, ON, Canada) using Hiperfect (Qiagen). The fast-forward protocol was followed as described by the manufacturer. RSK1 and RSK2 siRNA sequences were as previously described [38].

### Transient transfection

HCC1937 cells were seeded at a density of 4 × 10^5 ^in a six-well plate 24 hours prior to transfection. Cells were transfected with 2 μg plasmid DNA using 10 μl Lipofectamine 2000/well as per instructions, and were lysed at 24 hours. Plasmid constructs for RSK overexpression studies were empty vectors (pRK7 and pKH3), pKH3-avRSK1, pKH3-mRSK2, pRK7-Myr-avRSK1 and pKH3-avRSK1(K112/464R) (kinase-dead) as previously described [39]. The experiment was carried out three times.

### RSK2^-/- ^mouse embryo fibroblasts

Wild-type and RSK2^-/- ^mouse embryo fibroblasts (MEFs) were cultured as described previously [40], and were stimulated with EGF (10 ng/ml) and cell lysates collected at 5, 15, 30, 60 and 120 minutes (kind donation from Dr YY Cho, University of Minnesota, Austin, MN, USA). Two sets of samples were analyzed.

### Luciferase assay

SUM149 cells were plated in six-well plates (4 × 10^5 ^cells/well) and transfected with a luciferase construct containing the first 1 kb of the EGFR promoter (pER1) (kind gift from Alfred C. Johnson US National Cancer Institute, Bethesda, MD, USA – previously described in [1,41]). Cells were transfected with a total of 1.5 μg DNA using Lipofectamine 2000 (Invitrogen). To account for transfection efficiency, cells were co-transfected with a renilla-expressing plasmid (pRL-TK, 10:1 luciferase:renilla; Promega). After 18 hours, cells were treated with vehicle or SL0101 (50 μM) for 6 hours prior to harvesting in 1 × passive lysis buffer (Promega). Luciferase activity was measured and normalized to the renilla reading from the same sample.

### Chromatin immunoprecipitation

SUM149 cells (1 × 10^7 ^cells) were treated with vehicle, PD098059 (20 μM) or SL0101 (50 μM) for 6 hours. Crosslinks were established between protein and DNA following 15 minutes of incubation with 1% formaldehyde. Cells were washed and collected by centrifugation. Chromatin immunoprecipitation with anti-P-YB-1 antibody (gift from Dr P Mertens, University Hospital RWTH – Aachen, Aachen, Germany) was carried out as described previously [1,2]. The resulting DNA was amplified using the EGFR2a primers (previously described [1,2]).

## Results

### YB-1 is phosphorylated by the MAP kinase pathway

**Figure 1:**
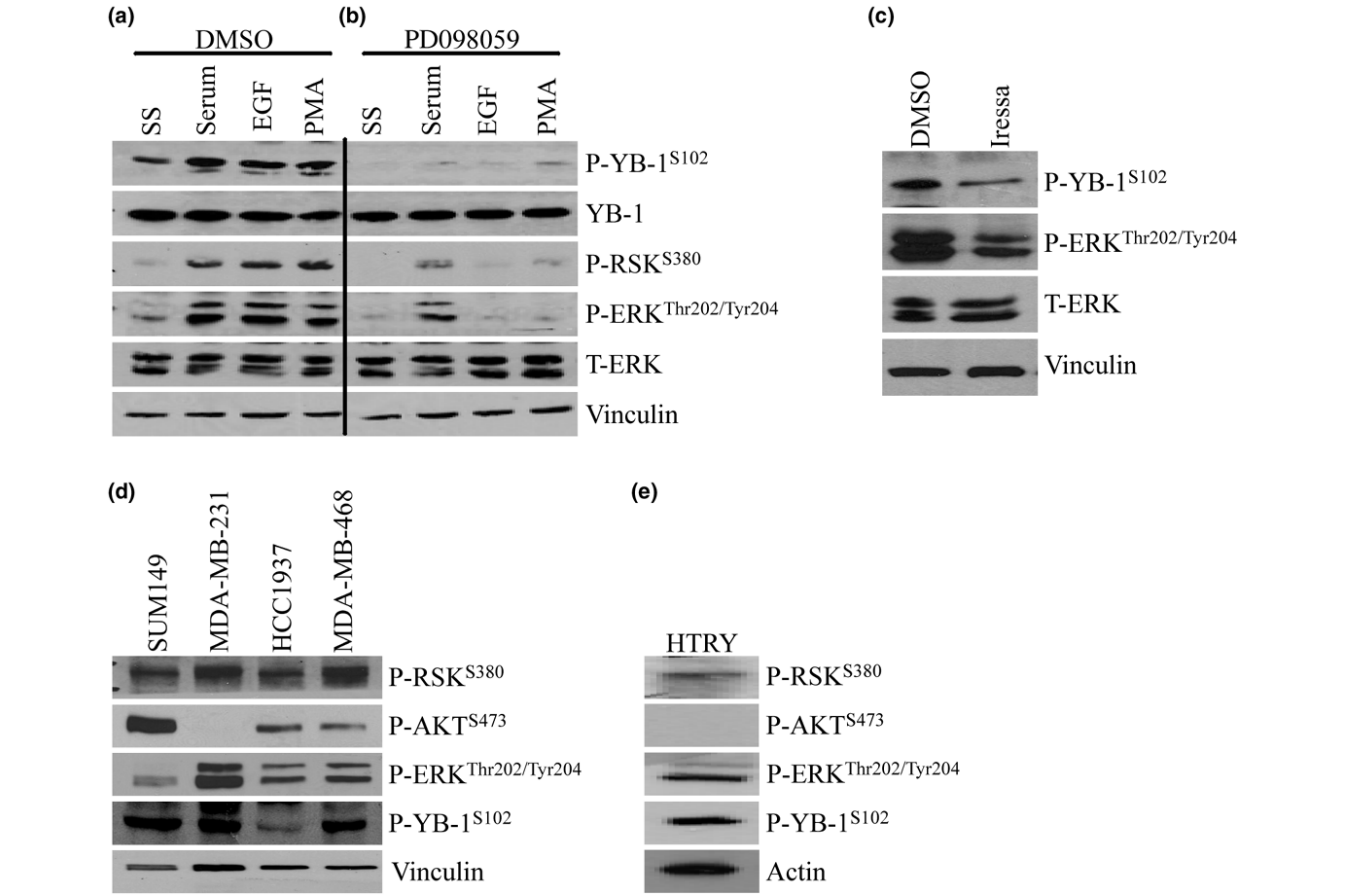
**Y-box binding factor-1 is phosphorylated by the MAP kinase pathway**. **(a) **Stimulation of SUM149 cells (SS) with serum, epidermal growth factor (EGF) and phorbal 12-myristate 13-acetate (PMA) (15 min) results in the phosphorylation of Y-box binding factor-1 (YB-1) at the serine 102 residue (S102). There is no change in total YB-1 levels. Phosphorylation of ERK indicates activation of the MAP kinase pathway. Total ERK and vinculin indicate equal loading. **(b) **Inhibition of MAP kinase signaling with PD098059 results in the loss of growth-factor induced P-YB-1^S102 ^(n = 3). **(c) **Treating SUM149 cells with Iressa (2 μM) results in a decrease in P-YB-1^S102^. **(d) **SUM149, MDA-MB-231, HCC1937 and MDA-MB-468 breast cancer cell lines were compared for expression level of P-RSK^S380^, P-AKT^S473^, P-ERK^Thr202/Tyr204 ^and P-YB-1^S102^. The MDA-MB-231 cells express high levels of P-YB-1 in the absence of P-AKT^S473^; however, they do express P-RSK^S380^. **(e) **Immortalized human mammary epithelial cells (HTRY) express P-RSK^S380^, P-ERK^Thr202/Tyr204 ^and P-YB-1^S102^, but not P-AKT. DMSO, dimethylsulfoxide.

While we have previously established that AKT can interact and phosphorylate YB-1^S102 ^[11], it is unclear whether other kinases are also able to perform this function. Serum-starved SUM149 cells were stimulated with 5% FBS/growth media, EGF or the tumour promoter PMA. All of these stimuli activated signaling through the MAP kinase/ERK pathway and led to the induction of P-YB-1^S102 ^(Figure 1a). The activation of the MAP kinase/RSK/YB-1 cascade was completely reversible by pretreating the cells with the MEK inhibitor PD098059 (Figure 1b). SUM149 cells secrete amphiregulin, resulting in activation of EGFR even in serum-free conditions [42]. We therefore also treated the cells with the EGFR inhibitor Iressa. As expected, inhibiting EGFR signaling with Iressa decreased P-YB-1^S102 ^(Figure 1c). By screening a panel of BLBC cell lines, we noted that YB-1 was activated at varying levels but, interestingly, the level of phosphorylation did not always correlate with the expression of P-AKT^S473^. RSK was activated in all cell lines including the MDA-MB-231 cells. These cells do not express P-AKT^S473^; however, the level of P-YB-1^S102 ^is comparable with that of the SUM149 cells, which express activated AKT as well as P-RSK^S380 ^(Figure 1d). Similarly, in the immortalized normal breast cell line HTRY, P-RSK^S380 ^is also elevated along with P-YB-1^S102^. These cells also do not express activated AKT (Figure 1e). It therefore appears that activation of the MAP kinase pathway can also lead to the induction of P-YB-1^S102^. This is of particular importance in BLBC given the role of EGFR signaling in this particular type of breast cancer.

### RSK phosphorylates YB-1 at serine residue 102

To further explore the role of the MAP kinase pathway in the phosphorylation of YB-1^S102 ^we next investigated the effect of modulating RSK, which lies downstream of ERK, either pharmacologically or genetically. Initially, using an *in vitro *kinase assay, we show that RSK1 and RSK2 are able to directly phosphorylate an YB-1 S102 peptide that mimics the region surrounding the S102 site (Table 1). The activity of RSK1 and RSK2 towards the YB-1 target peptide was 80% and 78% compared with the activity of these kinases towards the optimized positive control target, respectively (Table 1). Interestingly, this was greater than the activity of AKT1 towards the YB-1 target (7% of optimized control activity) (Table 1). The activity of AKT1, however, was still considered significant in this assay. Of note, the YB-1 target peptide was also phosphorylated by PKCα (Table 1). Weak RSK1 kinase activity was also detected when using flag-tagged YB-1 immunoprecipitated from stably expressing MCF-7 cells as a substrate (data not shown). In this case the salts required for the protein isolation compromised the level of activity.

**Table 1:** Activity of RSK1, AKT1 and PKCα against the Y-box binding factor-1(YB-1) serine 102 residue peptide compared with the optimized positive control substrate

Kinase	Activity against YB-1 peptide compared with control (%)
RSK1	80 ± 1.04
RSK2	78 ± 0.78
AKT1	7 ± 0.7
PKCα	19 ± 1.14

The p90 ribosomal S6 kinases RSK1 and RSK2 phosphorylated a peptide that mimics the serine 102 residue region of YB-1 with 80% and 78% efficiency compared with the positive control substrate, respectively. Both AKT1 and PKCα were also able to phosphorylate the YB-1 peptide – 7% and 19%, respectively, compared with the positive control. Activity for control substrates for each kinase is normalized to 100%. A change > 5% is considered highly significant in this assay.

**Figure 2:**
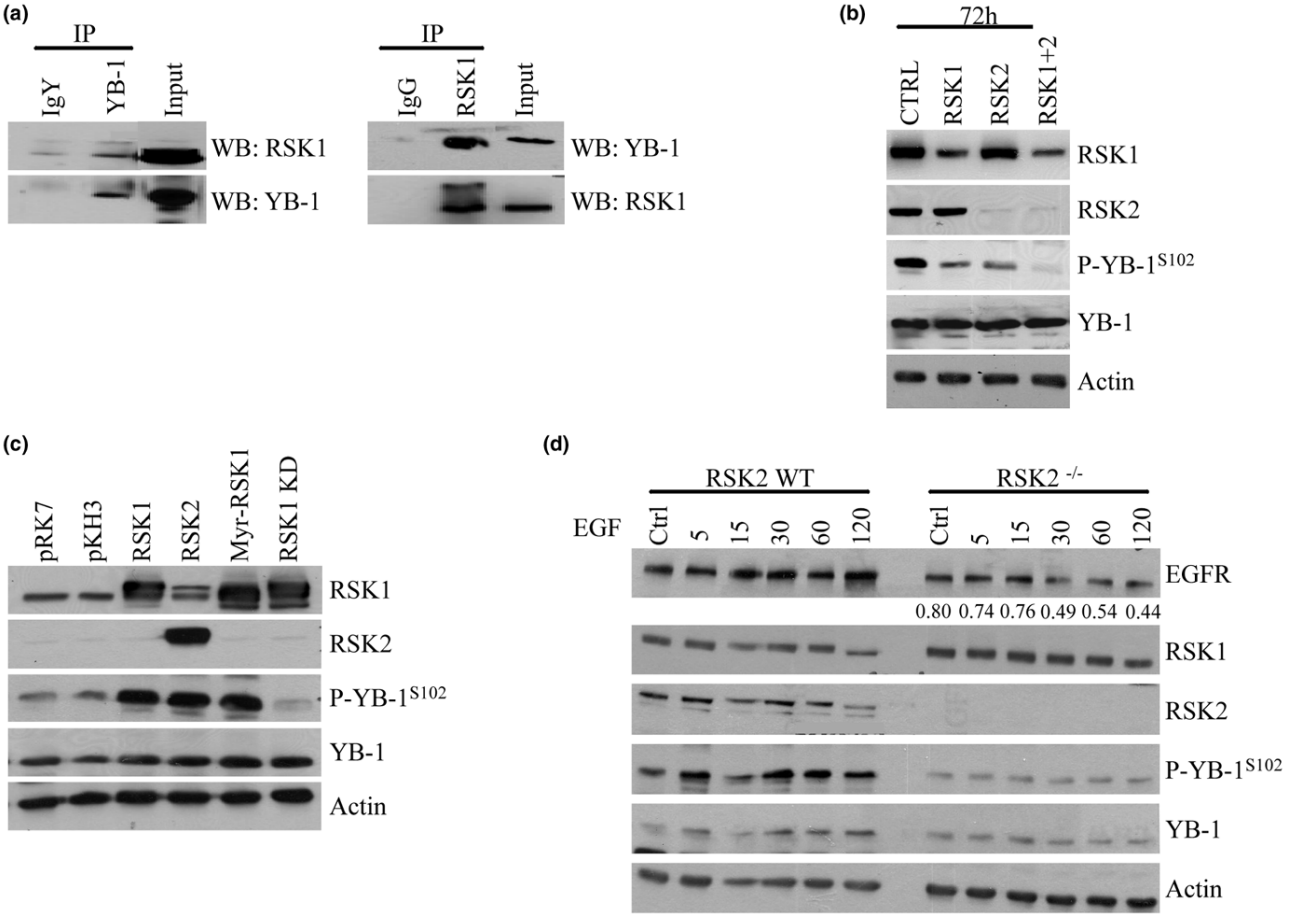
**p90 ribosomal S6 kinase phosphorylates Y-box binding factor-1 at the serine 102 residue**. **(a) **p90 ribosomal S6 kinase RSK1 is detected by immunoblotting following immunoprecipitation (IP) with Y-box binding factor-1 (YB-1) in SUM149 cells. Immunoprecipitation with IgY antibody was used to account for nonspecific binding (left). YB-1 is detected by pulling down and immunoblotting for RSK1. Immunoprecipitations performed with IgG antibody were used to account for nonspecific binding. Secondary detection was performed with horseradish peroxidase protein A (right). WB, western blot. **(b) **Transfection of SUM149 cells with RSK1, with RSK2 or with RSK1 and RSK2 siRNA for 72 hours reduces P-YB-1^S102 ^while total YB-1 remains unchanged. Actin acts as a loading control (n = 3). **(c) **HCC1937 cells transfected with RSK1 or activated RSK (Myr-RSK1) express elevated levels of P-YB-1^S102 ^compared with the control vector pKH3 (pRK7 for myr-RSK). A kinase-dead form of RSK (RSK1 KD) failed to induce P-YB-1^S102 ^and was comparable with the control (n = 3). **(d) **RSK2^-/- ^mouse embryo fibroblasts (MEFs) stimulated with epidermal growth factor (EGF) for a designated amount of time contain less P-YB-1^S102 ^than the wild-type mice. Epidermal growth factor receptor (EGFR) is also reduced, unlike RSK1 that was expressed at a comparable level in both sets of MEFs. The RSK2 immunoblot confirms the genotype of the mice, and actin was used a loading control. The relative expression levels of EGFR in the RSK2^-/- ^MEFs compared with wild-type MEFs are shown under the EGFR blot (n = 2). Ctrl, control.

We also found that, following immunoprecipitation of endogenous YB-1 from log-growing cells, RSK1 is present in the complex (Figure 2a, left). Similarly, by performing the reverse experiment, immunoprecipitation of RSK1, YB-1 was detected (Figure 2a, right). We were unable to determine an interaction of RSK2 with YB-1 due to a lack of suitable antibody for this application. Since RSK2 could not be detected following YB-1 immunoprecipitation, we believe the interaction between the two proteins maybe weaker. This prompted us to investigate the consequence of inhibiting RSK1 or RSK2 on YB-1 phosphorylation. Following suppression of RSK1 expression with siRNA for 72 hours, the level of P-YB-1^S102 ^was greatly reduced in SUM149 cells (Figure 2b). The loss of RSK2 also resulted in a decrease in YB-1 phosphorylation, although to a lesser degree than that by RSK1. Simultaneous knockdown of RSK1 and RSK2 produced an effect on the level of P-YB-1^S102 ^greater than either gene knockdown alone (Figure 2b). The levels of total YB-1 and actin remained unchanged (Figure 2b). In a complementary study, introducing exogenous RSK1, RSK2 or a constitutively active RSK1 (myr-RSK) for 24 hours induced P-YB-1^S102 ^in HCC1937 cells (Figure 2c) compared with cells transfected with the empty vectors pKH3 and pRK7 (myr-RSK empty vector). The kinase-dead RSK1 mutant, however, was unable to phosphorylate YB-1 at S102 (Figure 2c). Taking an alternative genetic approach, we turned to using MEFs that have a homozygous deletion for RSK2 [40]. Loss of RSK2 prevented the induction of P-YB-1^S102^ following EGF stimulation in a time-dependent manner, as compared with the wild-type MEFs (Figure 2d). ERK and RSK were still phosphorylated in response to EGF in the RSK2^-/- ^MEFs (data not shown). Interestingly, the YB-1 downstream target gene EGFR could be induced in the wild-type cells after 120 minutes; however, this was not the case in the RSK2^-/- ^cells (relative intensity of EGFR expression compared with wild-type cells at each time point given under blot) (Figure 2d).

**Figure 3:**
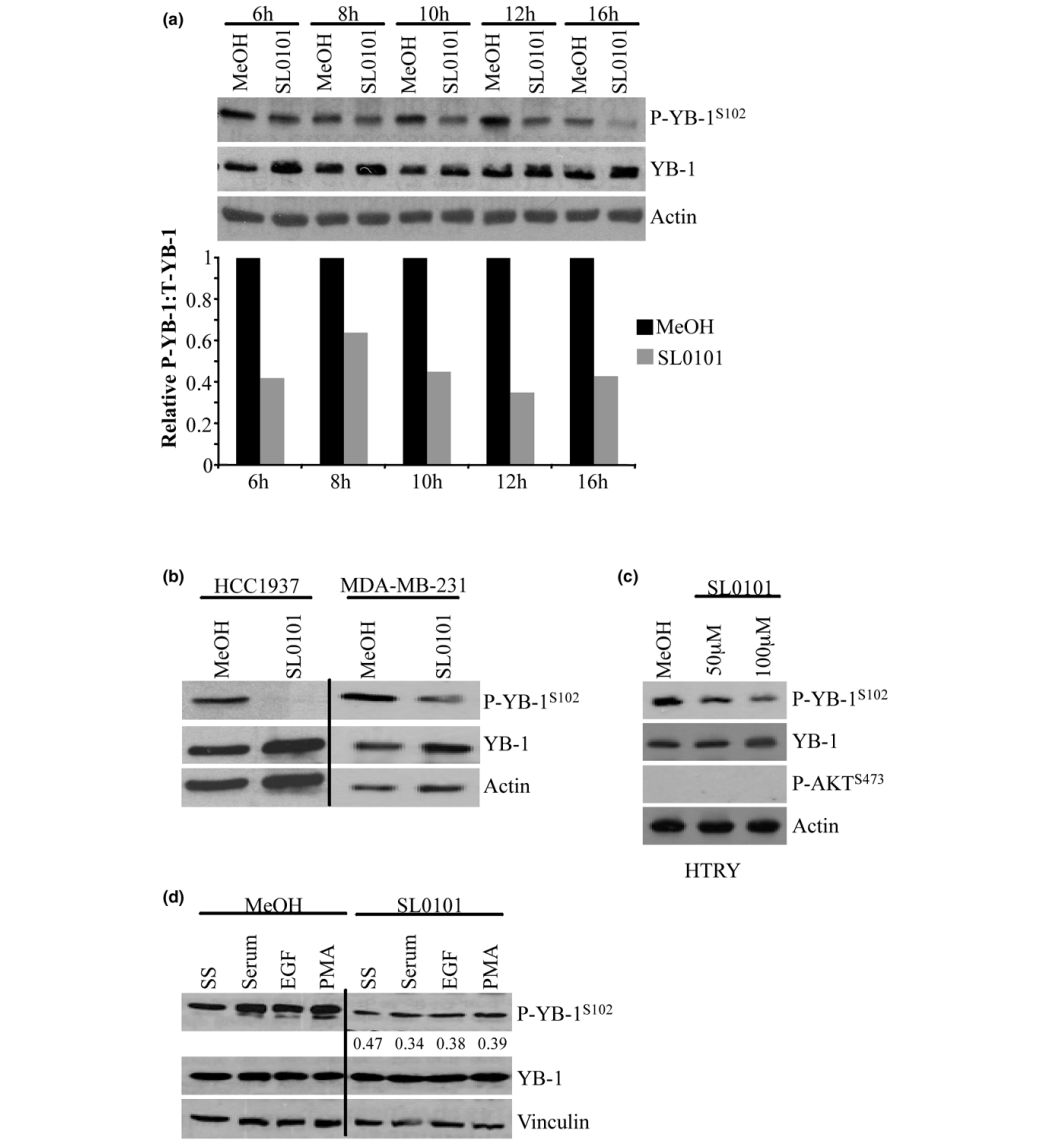
**Pharmacological inhibition of p90 ribosomal S6 kinase decreases Y-box binding factor-1 phosphorylation**. **(a) **Inhibition of p90 ribosomal S6 kinases RSK1/RSK2 with SL0101 (50 μM) in SUM149 cells resulted in decreased growth-factor induced P-YB-1^S102 ^over a time course of 6 to 16 hours. Immunoblot with densitometric analysis below. All changes are statistically significant (*P *< 0.01). **(b) **After 6 hours of treatment with SL0101 (100 μM), P-YB-1^S102 ^was decreased in HCC1937 and MDA-MB-231 cells while Y-box binding factor-1 (YB-1) remained constant. **(c) **Treatment of HTRY cells with SL0101 (100 μM) decreased P-YB-1^S102 ^in a dose-dependent manner. **(d) **Treatment of SUM149 cells with SL0101 (50 μM) reverses the phosphorylation of YB-1 induced by stimulation with growth factors. SL0101 has no effect on total YB-1. Vinculin was used as a loading control (n = 3). SS, stimulation of SUM149 cells; EGF, epidermal growth factor; PMA, phorbal 12-myristate 13-acetate.

We then used the RSK1/RSK2 specific inhibitor SL0101 [31] to confirm these findings. SL0101 was used at concentrations in line with previous studies in MCF-7 cells [31]. Following treatment of SUM149 cells with SL0101 (50 μM) for between 6 and 16 hours, we observed a reduction in P-YB-1^S102 ^at all time points whilst the YB-1 level remained constant (Figure 3a). This finding was confirmed in the HCC1937, MDA-MB-231 and HTRY cells treated for 6 hours with SL0101 (100 μM) (Figure 3b,c). Likewise, pretreating SUM149 cells with SL0101 prevented the stimulation of P-YB-1^S102 ^by serum, EGF or PMA after 6 hours compared with cells treated with the vehicle (methanol) control (Figure 3d). P-RSK^S380 ^is phosphorylated by the C-terminal kinase, and SL0101 inhibits the N-terminal kinase activity. One therefore cannot measure the effect of SL0101 by studying P-RSK^S380^.

### Inhibition of RSK functionally inactivates YB-1

**Figure 4:**
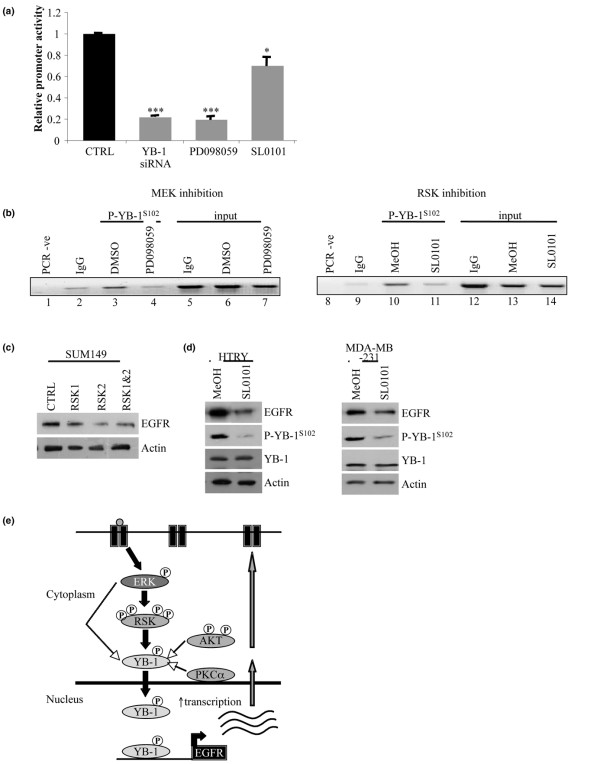
**Inhibiting p90 ribosomal S6 kinase modulates Y-box binding factor-1 transcription factor ability**. Inhibiting p90 ribosomal S6 kinase (RSK) modulates the ability of Y-box binding factor-1 (YB-1) to act as a transcription factor for epidermal growth factor receptor (EGFR). **(a) **EGFR promoter activity in SUM149 cells was reduced by 80% following knockdown of YB-1 or treatment with PD098059 (***P < 0.001) and by 30% (*P = 0.02) following treatment with SL0101 (50 μM). **(b) **Binding of P-YB-1^S102 ^to the EGFR promoter is reduced in the SUM149 cells following treatment with PD098059 (lane 4 compared with lane 3 (vehicle)) or SL0101 (50 μM) (lane 11 compared with lane 10 (vehicle)). IgG immunoprecipitation acts as a negative control. Input samples show amplification of the region in the cross-linked cells prior to immunoprecipitation (n = 2). DMSO, dimethylsulfoxide. **(c) **Transfection with RSK2 siRNA for 72 hours led to a decrease in EGFR expression in SUM149 cells. **(d)** Treatment of immortalized breast mammary epithelial cells (HTRY) (10 hours) or MDA-MB-231 cancer cells (12 hours) with SL0101 results in loss of P-YB-1^S102 ^and a concomitant reduction in EGFR. **(e) **Model demonstrating the positive feedback loop generated on the activation of YB-1 by EGFR. Ligand binding to the receptor activates signaling pathways such as MAP kinase, resulting in the phosphorylation of RSK. Once the kinase is fully activated, it phosphorylates YB-1 at S102 – subsequently allowing YB-1 to play a role in promoting translation and to enter the nucleus as a transcription factor. AKT and PKCα can also activate YB-1 following growth factor stimulation. On binding to inverse CAAT boxes, YB-1 promotes the transcription of genes such as EGFR – resulting in increased surface expression of the receptor. Ctrl, control.

We have previously established the importance of phosphorylation of YB-1^S102 ^for its transcriptional activity in breast cancer [2], and in particular the regulation of EGFR in BLBC. Firstly, we performed a reporter assay using a 1 kb EGFR–luciferase construct that contains an YB-1 binding site at -968 base pairs [1]. Knocking down YB-1 with siRNA or inhibiting signaling with PD098059 decreased the EGFR promoter activity by ~80% (*P *< 0.001) (Figure 4a), while inhibition further downstream with the RSK inhibitor SL0101 decreased EGFR reporter activity by 30% (*P *= 0.02) (Figure 4a). Consistent with this observation, PD098059 and SL0101 prevented P-YB-1(S102) from binding to the EGFR promoter based on chromatin immunoprecipitation (Figure 4b).

Inhibition of RSK2 by siRNA in SUM149 cells (Figure 4c) led to a decrease in EGFR expression. This downregulation was mirrored in HTRY and MDA-MB-231 cells following treatment with SL0101 (Figure 4d); densitometric analysis for MDA-MB-231 gave a 35% decrease. We thereby conclude that there is a feed-forward signaling pathway in BLBC where EGF binds to the EGFR, which in turn leads to activation of the MAP kinase/RSK pathway resulting in phosphorylation of YB-1 at S102. Activated AKT and PKCα also have the ability to activate YB-1. Following this, P-YB-1^S102 ^binds to and transactivates the EGFR gene, further fueling the growth potential of BLBC (Figure 4e).

## Discussion

We reveal for the first time that phosphorylation of YB-1 at the S102 location is not only carried out by the PI3K cascade but that signaling through the MAP kinase pathway can also activate this transcription factor. This is particularly relevant in BLBC, where EGFR is overexpressed in over one-half of the cases. More specifically it is the serine/threonine kinases RSK1 and RSK2 that are able to phosphorylate YB-1 at the key S102 residue in BLBC cells. Not only do we identify RSK1 and RSK2 as proteins that can directly interact and phosphorylate YB-1, but they have a much greater efficiency towards the target than AKT1 does. In fact, we also identified PKCα as having greater kinase activity towards YB-1 than AKT1, a finding that warrants future investigation. Phosphorylated RSK is also expressed in cell lines where we find abundant P-YB-1^S102 ^and a lack of active AKT; in particular the MDA-MB-231 cells and the immortalized human mammary epithelial cells, where we were unable to detect any P-AKT^S473^. The RSK1/RSK2-specific inhibitor SL0101 [31,43], as well as RSK1-targeted or RSK2-targeted siRNA, were able to reduce the phosphorylation of YB-1 at S102 even following induction by the classic tumour promoter PMA. Furthermore, we observed a reduced level of P-YB-1^S102 ^in RSK2^-/- ^MEFs. Finally, inhibition of RSK prevented P-YB-1^S102 ^binding to the EGFR promoter and ultimately reduced the protein expression of this receptor tyrosine kinase.

Our data are consistent with a recent study by Hoadley and colleagues reporting that EGFR and genes encoding components of the MAP kinase pathway were associated with the basal-like subtype, while AKT1 was not [44]. Interestingly, we found in our four BLBC cell lines that ERK2 expression was predominantly expressed over ERK1. This is in concordance with the analysis observed by Hoadley and colleagues, which shows expression of ERK2 was increased in the BLBC cluster, but this was not the case for ERK1 [44]. It is thus conceivable that ERK2 may activate RSK and therefore YB-1 in basal-like tumours. In this context it is also of interest that we in fact find YB-1^S102 ^to be a better substrate for RSK1 and RSK2 than AKT1. ERK2 may also directly phosphorylate YB-1 and therefore promotes its ability to transactivate target genes. In support of this idea, ERK2 promotes the transactivation of vascular endothelial growth factor by YB-1 [45]. This occurs when ERK2 phosphorylates the N-terminal region of YB-1; the region of the protein required for gene transactivation [16]. More recently, we identified a putative ERK phosphorylation site at serine 36 in this same region of the protein using Motif Scanner [22]; however, this has not been validated experimentally. While speculative at this point, if ERK does phosphorylate the transactivating domain of YB-1 this could explain why inhibiting ERK activity with PD098059 was better than SL0101 at suppressing EGFR reporter activity. In theory, inhibiting ERK2 would directly decrease phosphorylation of YB-1 at S36 at the N-terminal and indirectly block RSK from phosphorylating S102. These studies indicate that the MAP kinase pathway would have broad effects on YB-1.

While the emphasis of this study has been on BLBC, EGFR is equally important in promoting growth signals in other types of breast cancer. For example, EGFR forms heterodimers with Her-2 to engage signaling through either the MAPK or AKT pathways, which perhaps also involves RSK as well as AKT. This obviously could be important in stimulating the growth of breast cancer cells harboring amplified Her-2. Beyond breast cancer, we suspect that the relationship between RSK and YB-1 could be important in other malignancies. A study by Cho and colleagues demonstrated that RSK2 was a transforming gene, since stable expression in skin cells increased the colony number in anchorage-independent conditions [40]. Conversely, knockdown of RSK2 reduced colony formation even in the presence of constitutively active oncogenic Ras [40]. Other studies implicate RSK2 in transmitting the prosurvival and proliferative signals from oncogenic mutant receptor tyrosine kinase FGFR3 in multiple myeloma, resulting in cell transformation [28,46]. Interestingly, YB-1 has been implicated in the survival and progression of multiple myeloma cells – the expression correlating with rapid proliferation and poor differentiation [47]. We therefore postulate a model where RSK is activated through aberrant tyrosine kinase signaling, resulting in the subsequent phosphorylation of YB-1. In this way the cell will be influenced by any number of a diverse collection of genes that YB-1 has been shown to regulate, such as EGFR [1,14], Her-2 [14], topoisomerase II [5,7] and the multidrug resistance gene [48,49]. This regulation in fact may result in a positive feedback loop in the case of genes such as EGFR.

Beyond regulating transcription, YB-1 also promotes translation, alternative splicing, RNA transport and DNA repair [17,18,50-52]. Whether phosphorylation of YB-1 at S102 is important for these events is not known. Interestingly, RSK itself promotes translation through several mechanisms [23,39,53,54]; therefore, the role of these two proteins acting together in this process needs to be further investigated.

## Conclusion

We conclude that RSK1 and RSK2 are able to phosphorylate YB-1^S102^, providing a newly described mechanism whereby this transcription factor is activated in breast cancer. In fact, RSK activates YB-1 more effectively than AKT and may therefore be the major facilitator of YB-1 function in BLBC. Interest in developing small molecules against RSK has increased over the past 2 years, and we believe this could be an important opportunity for therapeutic intervention. As RSK has never before been associated with BLBC, we therefore introduce a new mechanistic understanding and potentially a therapeutic strategy for treating this aggressive disease.

## Abbreviations

BLBC: basal-like breast cancers; DMEM: Dulbecco's modified eagle medium; EGF: epidermal growth factor; EGFR: epidermal growth factor receptor; ELB: egg lysis buffer; ERK: extracellular signal-regulated kinases; FBS: fetal bovine serum; MAP: mitogen-activated protein; MEF: mouse embryo fibroblast; PKCα: protein kinase C alpha; PMA: phorbal 12-myristate 13-acetate; RIPA: radio immunoprecipitation assay; RSK: p90 ribosomal S6 kinase; S102: serine 102 residue; siRNA: small interfering RNA; YB-1: Y-box binding factor-1.

## Competing interests

The authors declare that they have no competing interests.

## Authors' contributions

ALS drafted the manuscript and performed experiments unless stated otherwise. CJF and CD made the phospho-YB-1^S102 ^antibody. AHD performed Flag-YB-1 for the kinase assay. YL carried out the Iressa treatment. YYC and ZD provided samples from EGF-stimulated RSK2^-/- ^MEFs. IMB provided the HTRY cells. PPR provided the RSK constructs and conceptual suggestions. SED conceived the studies and was involved in editing the manuscript.
